# Positive and Negative Factors That Influence Health Care Faculty Intent to Engage in Interprofessional Education (IPE)

**DOI:** 10.3390/healthcare7010029

**Published:** 2019-02-16

**Authors:** Maria Olenick, Monica Flowers, Teresa Muñecas, Tatayana Maltseva

**Affiliations:** Nicole Wertheim College of Nursing and Health Science, Florida International University, 11200 SW 8th Street, AHC3-Rm 329, Miami, FL 33199, USA; mflower@fiu.edu (M.F.); tmunecas@fiu.edu (T.M.); bogopolt@fiu.edu (T.M.)

**Keywords:** interprofessional, interdisciplinary, health care faculty, barriers

## Abstract

*Background:* This study explored the positive and negative factors that influence interprofessional education (IPE) implementation in health care education programs across the United States. *Methods:* The study sample consisted of 439 (response rate 8.4%) health care faculties from seven health care professions (nursing, medicine, pharmacy, physical therapy, occupational therapy, physician assistant, and social work) who were asked what the positive and negative factors are that influence their decisions to engage in IPE. *Results:* Three positive categories and two negative categories concerning factors that influence health care faculty (HCF) intent to engage in IPE emerged. The three major categories of positive factors emerged as patient care, which was mentioned by 196 subjects or 54% of the sample, student learning, which was mentioned by 157 subjects or 43% of the sample, and health care teams, which was mentioned by 88 subjects or 24% of the sample. The two major categories of negative factors emerged as scheduling and coordination, which was mentioned by 230 subjects or 63% of the sample, and discipline culture which was mentioned by 103 subjects or 28% of the sample. *Conclusion:* This study contributes to the body of knowledge concerning the factors that influence effective IPE implementation. Discussion is provided regarding the positive and negative categories that emerged and how they influence effective IPE delivery.

## 1. Introduction

Interprofessional education (IPE) is an experiential learning and socialization collaborative process between and among health care disciplines [1]. Olenick et al.’s concept analysis and the operational definition of IPE for the purpose of this research defines IPE as follows: “IPE occurs when two or more members of a health care team, who participate in either patient assessment and/or management, learn with, from, and about each other as they collaboratively focus on patient-centered care and achieving optimal health outcomes. In IPE, knowledge and value sharing occur within and across disciplines” [1] (p. 6). Effective and collaborative IPE diminishes negative attitudes and stereotypes; IPE promotes effective relationships and optimal patient-focused care and safety [2].

Traditionally, health care disciplines were taught within their own silos where they were grouped within their own ontologic perspectives, where they related within a hierarchy and where they were subdivided according to similarities and differences among them. Interprofessional education (IPE) is an entirely new way of considering education cooperatively among various health care faculty (HCF) disciplines. The Institute of Medicine’s (IOM) landmark publication in 2011 [3] clearly recommends and encourages IPE for collaboration and leadership in health care delivery and redesign for all health care disciplines, but specifically for the future of nursing. HCF perceptions about skills, abilities, information, emotions, and the perceptions about situational and environmental factors contribute to the way HCFs act or react in relation to IPE. 

In this study, HCFs in the United States (U.S.) identified the positive and negative factors that have or would have encouraged or prevented them from engaging in IPE. Nurses (NU) and medical doctors/physicians (MD) reported the lowest percentages of negative factors about IPE. Occupational therapists (OT) and physician assistants (PA) reported the highest percentages of negative factors about IPE. 

Negative factors impact the implementation of IPE. The identification of negative factors that impede IPE in the U.S. is the first step in overcoming them. Shifts in beliefs regarding disciplinary territory, entrenched tradition, and turf concerns will take time, energy and displays of improved cooperative efforts between and among disciplines before more cooperative IPE begins to effectively emerge. 

### Research Questions

Subjects in this study were asked two open-ended questions concerning the positive and negative factors that influence their intent to engage in IPE:

(1) What are the positive factors that have influenced/would influence you to engage in IPE?
(2) What are the negative factors that have prevented/would prevent you from engaging in IPE?

## 2. Methodology

### 2.1. Methods

A qualitative research approach using content analysis was conducted. Frequency counts through the Survey Monkey™ text analysis feature were utilized to identify the frequency of word occurrence initially. Once those words were identified, content analysis was utilized to distinguish among the concepts and code text. The emergence of words and phrases was examined for the meaning and context in which they emerged.

### 2.2. Sample Selection and Size

Health care faculties with publicly available email addresses were identified from randomly selected U.S. health care programs of colleges and universities, according to the four U.S. census bureau regions (Northeast, Midwest, South, and West) [4]. Five thousand, two hundred and twenty-four emails with research questions were sent out to faculties at 274 schools from a stratified random sample of nursing, medicine, pharmacy, physical therapy, occupational therapy, physician assistant, and social work programs across the U.S. A final total of 439 replies were received and analyzed, which means that an 8.4% response rate was achieved. Health care programs in seven different disciplines were included based on the following criteria:

1. Baccalaureate and higher degree programs only
2. Nursing (NU) programs accredited by the Commission on Collegiate Nursing Education (CCNE) or the National League of Nursing Accrediting Commission (NLNAC)
3. Allopathic medical (MD) schools accredited by the Liaison Committee on Medical Education (LCME)
4. Pharmacy (PH) schools accredited by the Accreditation Council for Pharmacy Education (ACPE)
5. Physical therapy (PT) schools that were accredited by the Commission on Accreditation in PT Education (CAPTE)
6. Occupational therapy (OT) schools that were accredited by the Accrediting Council for OT Education (ACOTE)
7. Physician assistant (PA) programs that were accredited by the Accreditation Review Commission for the Physician Assistant (ARC-PA)
8. Social work (SW) programs that were accredited by the Council on Social Work Education (CSWE).

### 2.3. Data Collection

Institutional Review Board (IRB) approval was attained by this researcher from Widener University, Chester, PA. Email messages that provided the study purpose, procedures, risks, benefits, alternatives, costs, compensation, confidentiality, right to withdraw, principle investigator contact information, and a link to the online survey were sent to HCFs. In an attempt to achieve a predicted response rate of 80%, Dillman’s Tailored Design Method (TDM) was used [5].

Participation in the study was voluntary. HCFs with publically available email addresses from the seven health care disciplines were identified from 10–20% of randomly selected health care programs within the four census bureau U.S. regions. Those HCFs received emails with an online Survey Monkey™ link. 

No names were attached to the online submitted surveys, so subject anonymity and confidentiality were maintained. Submission of the online survey implied informed consent. 

### 2.4. Research Methodology

Frequency counts through text analysis were utilized to identify the frequency of word occurrence initially. Once those words were identified, conceptual analysis was utilized to distinguish among the concepts and code text. Relational analysis determined the meanings that emerged and examined words and phrases that revealed positive and negative factor categories from HCF responses. 

## 3. Results

The analysis of the positive and negative factors affecting IPE implementation from the final sample (*n* = 439) is discussed, summarized, and presented here. 

### 3.1. Positive Factors Influencing Engagement in IPE

Twenty-six initial categories of positive factors that influenced engagement in IPE were identified through frequency counts by the software. Categories that were identified through use of this feature included the 15 words or phrases that were most commonly used by respondents. The details are presented in Table 1. This first step of text data analysis only counted the frequency of words, not the number of subjects who used the words.

“Care”, “Students”, “Patient”, “Learning”, and “Team” were the five categories counted most frequently as positive factors. Nine other categories were identified that occurred less than 10% of the time, while 12 categories were mentioned less than 2% of the time.

### 3.2. Negative Factors Influencing Engagement in IPE

The negative factors influencing engagement in IPE were also identified through the same Survey Monkey™ text analysis feature. There were 28 initial categories that were identified with 17 words or phrases being used by respondents most often. The details are presented in Table 2. This first step of text data analysis only counted the frequency of words, not the number of subjects who used the words.

“Scheduling”, “Professional”, “Students”, “Disciplines”, and “Coordination” were counted most frequently as negative factors. Twelve other categories were identified that occurred from 5% to 2% of the time. An additional 12 identified categories were cited by less than 2% of the participants.

### 3.3. Category Reduction

Electronic content analysis was used to reduce the number of initial categories [6]. Quotes were placed in excel spreadsheets and sorted according to the Survey Monkey™ categories. The quotes were then highlighted and re-highlighted with different colors in an effort to collapse the many categories into fewer major categories and describe those categories. Examples of HCF quotes about the positive and negative factors influencing their intent to engage in IPE are presented in Table 3 and Table 4, respectively. 

The three major categories of the positive factors emerged as patient care, which was mentioned by 196 subjects or 54% of the sample, student learning, which was mentioned by 157 subjects or 43% of the sample, and health care teams, which was mentioned by 88 subjects or 24% of the sample. Quotes that support the patient care major category include: “my experience has been that the potential exists for improved patient outcomes”, “more complete care of the patient”, “holistic care of the patient”, “improved patient care by a team able to communicate”, and “as healthcare is a team concept, positive patient outcomes improve when the team members work together”. Quotes that support the student learning major category include: “potential for enhanced student learning”, “opportunities for students to be exposed to information outside our discipline”, “learn from other health professionals”, “motivate students to better understand other health professions”, and “the opportunity to offer students a positive learning experience”. Quotes that support the health care teams major category include: “early role modeling of working in healthcare teams”, “practicing teamwork and communication; learning to respect what each discipline brings to patient care”, “understanding the benefit of learning as a team since healthcare professionals work as a team to care for patients”, “I have played several roles in a healthcare team through my past experience and understand the importance of knowing and utilizing the full capabilities of all team members for the benefit of the patient”, and “we must embrace IPE and the team approach”.

The two major categories of the negative factors emerged as scheduling and coordination, which was mentioned by 230 subjects or 63% of the sample, and discipline culture, which was mentioned by 103 subjects or 28% of the sample. Quotes that support the scheduling and coordination major category include: “overcoming scheduling difficulties”, “scheduling challenges, resistance from other departments/professions”, “there is often not a time to coordinate the team concept particularly in academic setting where the colleges are on different schedules”, “coordination can be difficult if all faculty members are not engaged”, “scheduling difficulties―trying to get all of the professions together at a particular time”, “no time or incentive to coordinate with other healthcare professions”, and “difficulty in scheduling issues with various disciplines”. This is consistent with the literature as revealed by Al Achkar, Hanauer, Colavecchia, and Seehusen [7], where they found that more than half of both faculty and residents in a graduate medical residency program felt that lack of time is a barrier to IPE. 

Quotes that support the discipline culture major category include: “turf wars”, “discipline elitism”, “territorial disputes on common areas of practice”, “the remaining attitudes of some professionals that they are the final authority in the healthcare inertia”, “I guess the main factor would be the level of cooperation”, “stronger focus on other professionals”, and “people being territorial”.

### 3.4. HCF Percentages of Positive and Negative Factors Reported

Electronic content analysis was conducted to determine the percentages of HCFs who offered text responses for the positive and negative factors influencing their intent to engage in IPE (Table 5). 

Responses were placed in excel spreadsheets and sorted according to the seven HCF categories. The responses were then color-coded for each HCF group and the percentages of HCF group responses were calculated. MDs had the lowest response rate for both positive factors and negative factors at 76.3%. OTs had the highest response rate for both positive factors and negative factors at 90%. The percentages for the identified positive and negative factors ranged from 76.3% to 90%. NUs responded at a rate of 81.2% for positive factors and a rate of 79.1% for negative factors. HCF text responses to two open-ended questions concerning the positive and negative factors influencing their intent to engage in IPE were analyzed. Three positive and two negative major categories were identified and exemplars of HCF statements were presented. The percentages of positive and negative factors identified by each of the seven HCFs were computed. 

**Positive factors.** Factors in this category were organized into three major categories. Subject responses most often identified improved patient care, student benefits, and health care teams as the most common factors that influenced their intent to engage in IPE which is supported by the literature [2]: to improve collaboration, to improve communication, to improve patient safety, to improve health care quality, and to improve attitudes toward teamwork.

*Category One: Patient care*. When the words patient and care occurred in the quotes, they most frequently occurred together and most commonly referred to improved patient care or optimal patient care. According to the responses, subjects in this study believed that IPE contributes to better, patient centered, and quality health care for patients. 

The positive factors associated with IPE are supported in the literature. The available literature identifies improved patient outcomes as a result of IPE and or interprofessional teams. Several studies confirm that interprofessional teams improve patient outcomes [8,9] and that patients were more satisfied with their care because of information sharing [10]. The non-empirical literature also cites improved patient outcomes as a benefit of IPE; however, there is a large gap in the empirical literature to support this claim. Despite the lack of scientific evidence that patients benefit from IPE, it was clear from the quotes in this study that HCFs continue to believe this is true.

*Category Two: Student learning*. When the words student and learning occurred in the quotes, they most frequently occurred in the context of student benefits, learning opportunities, learning other perspectives, and learning experiences. According to the responses, subjects in this study believed that IPE contributes to overall enhanced student learning. Optimal student learning is central to the vision and goals of HCFs and educational institutions. Studies have shown improved clinical decision-making skills, and knowledge scores among medical students after IPE as compared to a control group not receiving IPE [11].

*Category Three: Health care teams*. When the words health care and team occurred in the quotes, they most frequently occurred in the context of integrated health care teams, working in teams, and preparation for actual practice as health care providers since providers function in teams. According to the responses, subjects in this study believed that IPE contributes to embracing a team approach to health care for the betterment of patient outcomes. Research supports that interprofessionalism increases quality, safety, patient satisfaction, and decreases length of stay [12].

The Association of American Medical Colleges (AAMC) requires physicians to establish interprofessional collaboration and teamwork competencies, which students must perform independently prior to enter residency [13]. Effective teamwork in health care enhances safety, efficiency, and quality of patients’ care outcomes [13].

**Negative factors.** Factors in this category were organized into two major categories. Subject responses most often identified scheduling and coordination, and discipline culture as the most common factors that prevent them from engaging in IPE. 

*Category One: Scheduling and coordination*. According to the responses, the coordination of schedules with other health care professional programs and students can be a daunting task especially when territorial, turf, and non-optimal cooperation between disciplines exists. According to the responses, subjects in this study believed that the time, work, effort, challenges, difficulties, and logistics in combination with the levels of cooperation between professionals in differing disciplines is the number one reason why IPE is not implemented effectively. 

The negative factors identified in this study are consistent with reports in the literature where logistics, including timetabling, geography, and physical space were identified as factors that presented barriers to IPE implementation. Al Achkar et al. [7] and Townsend, Pisapia, and Rassaq [14] identified time and space as barriers to IPE. Awareness of the factors that inhibit IPE will allow for the generation of solutions to known problems or issues. Scheduling and coordination are logistical challenges. However, they are not impossible for schools’ administrators and general HCFs to overcome if they wish to engage in IPE.

*Category Two: Discipline culture*. According to the responses, territorial issues, and attitudes between and toward other disciplines were the main factors that prevented HCFs from effectively engaging in IPE. Subjects in this study cited territorial disputes, the lack of cooperation between disciplines, and discipline elitism as factors impeding IPE. Shagrir [15] states that, with regard to collaborations within institutions where participants work, most (87%) stated that they prefer to collaborate with colleagues who hold similar roles to theirs. Issues such as ultimate accountability for patient care, reimbursement for patient care services, compensation for IPE efforts, and territorial concerns perpetuate miscommunications, disagreements, and disrespect between health care providers. The natural overlap of health care providers should be nurtured and supported in education and in the health care industry if there is ever to be a shift in discipline culture. 

## 4. Discussion

The negative factors reported in this qualitative study may prevent health care professional groups from cooperating and collaborating. Havyer et al. [13] recommended to maximize health care professionals’ involvement in patient care teams, with a focus on IPE assessment and debriefing, in order to gain cultural interprofessional teamwork behaviors. The new knowledge of the positive and negative factors that affect IPE, gained through this study, informs current broad knowledge of IPE. These findings raise awareness of the barriers affecting effective IPE implementation. Overcoming barriers to effective IPE and maximizing the positive factors that contribute to IPE for collaboration and leadership in health care delivery follow The Institute of Medicine’s (IOM) landmark publication in 2011 [3] which clearly recommends and encourages IPE.

### 4.1. Based on the Findings of This Study

1. Most HCFs identify enhanced patient care and improved patient outcomes as positive factors relating to IPE that influence them to engage in IPE.
2. Most HCFs identify scheduling issues as the primary negative factor preventing them from engaging in IPE.

### 4.2. Recommendations for Future Research and Future IPE Strategies

As a result of this study, the following recommendations for future research and strategies for future effective IPE delivery are suggested:

1. Identify best practices to eliminate existing barriers and negative factors related to IPE.
2. Identify best practices to reinforce positive factors to influence effective IPE implementation.

## 5. Conclusions

In IPE, the status of the health care environment can become a shared, collaboratively focused entity where members of highly integrated teams deliver optimal patient care. IPE may decrease fragmentation in health care delivery, and relinquish hierarchies, misperceptions, and miscommunications. IPE contributes to a holistic approach where all health care providers recognize one another’s contributions. Barriers to IPE implementation are evident based on the negative factors that HCFs in the U.S. present in this research. These barriers may be overcome by addressing the logistical challenges related to scheduling and coordination, and nurturing interprofessional relationships. 

## Figures and Tables

**Table 1:** Positive factor category occurrence.

Category	Number of Times the Word or Phrase Occurred	Percentage of Times the Word or Phrase Occurred
Care	103	28%
Students	89	24%
Patient	77	21%
Learning	65	17%
Team	58	15%
Education	30	8%
Perspectives	28	7%
IPE	27	7%
Teaching	21	5%
Support	16	4%
Respect	16	4%
Engaging	12	3%
Interprofessional	11	3%
Medical	11	3%
Programs	10	2%

IPE: Interprofessional Education.

**Table 2:** Negative factor occurrence.

Category	Number of Times the Word or Phrase Occurred	Percentage of Times the Word or Phrase Occurred
Scheduling	67	18%
Professional	39	10%
Students	36	9%
Disciplines	29	7%
Coordination	24	6%
IPE	22	5%
Faculty	21	5%
Health Care	17	4%
Support	16	4%
Course	13	3%
Constraints	13	3%
Departments	12	3%
Curriculum	12	3%
Team Members	10	2%
Attitudes	10	2%
Limited	9	2%
Take	8	2%

**Table 3:** Quotes that support positive factors.

Discipline	Quote
NU	“IPE teams are learning to collaborate and make less errors in patient care. Students are also learning about roles and capabilities of students from other disciplines.”
NU	“Broader perspective and better outcomes.”
NU	“Students would learn the importance of collaboration with other disciplines; also recognize what those disciplines do―what their role is in the care of patients as well as the other disciplines understand what nursing does.”
NU	“It’s how health care world operates. I am an NP and have been part of health care teams in that role, and even in the hospital, we worked together, even if it wasn’t overtly described as that.”
NU	“Growth, positive attitudes and mutual understanding, benefits to patients.”
MD	“Accelerated learning, better team-based care, better development of care teams of this decade. More enthusiasm in the clinical learning environment, better integrated learning across professions (horizontal spread of curricular content.”
MD	“Perceived improved patient outcomes.”
MD	“I have always collaborated with other disciplines to care for my patients. Doesn’t everyone?”
MD	“More complete care of the patient.”
MD	“Interdisciplinary teamwork is essential in my field―Geriatric Medicine.”
PH	“It is important that all health professionals learn to work together and see each other’s points of view so that patients benefits and egos do not get in the way. There is too much waste in education and health care due to turf battles.”
PH	“Ultimately, better patient care. Learning from other disciplines. Mimics actual practice. Helps remove myths/misperceptions about other professions.”
PH	“Development of teamwork and respect between the different students.”
PH	“Necessary to teach students to work together as a team. Shows them each their role and how they work collaboratively.”
PH	“The chance to be part of a team positively affecting patient care.”
PT	“Patient outcomes.”
PT	“Broadens the scope of students’ expertise in treating patients.”
PT	“The opportunity to offer students a positive learning experience. The opportunity to offer improved health care services to clients and patients.”
PT	“Student learning outcomes. Ultimately patient care outcomes.”
PT	“The real world requires health care professionals to work in teams―therefore we owe it to our students to give them some preparation relative to this.”
OT	“My experience has been that the potential exists for improved patient outcomes at lower cost and with better cohesiveness for those served. All team members gain skills in clinical procedures/activities and in leadership/organization. These factors compel me to keep this model on my radar as I engage in program development.”
OT	“Getting to know coworkers better; higher quality patient care; more appropriate use of other professions expertise.”
OT	“Greater understanding of our role in the team and to pass this understanding along to our students.”
OT	“Exposing students to the real world!”
OT	“I can learn from others’ expertise and perspectives. IPE enhances quality of care.”
PA	“The students find the experiences more engaging. They get more realistic learning than traditional classes.”
PA	“Benefit to students.”
PA	“I feel this would ultimately benefit the patient with better care. Care teams would be more knowledgeable about other professionals on the team.”
PA	“Mutual benefit. Interprofessional respect.”
PA	“Critical to my profession. Better outcomes. Good modeling of IP teams for students.”
SW	“Social workers engage with other professionals in their jobs and must be able to interact effectively with other healthcare professionals. Therefore, students need to learn how to work across disciplines.”
SW	“Opportunity to coordinate various perspectives, increased effectiveness in teaching by broadening information beyond my own expertise, role modeling interdisciplinary efforts/collaboration, and teaching comprehensive care for future practitioners.”
SW	“Superior student training.”
SW	“Preparing students to be productive members of healthcare teams.”
SW	“There is a need to share information across disciplines.”

NU: Nursing; MD: Medical Doctor; PH: Pharmacy; PT: Physical Therapist; OT: Occupational Therapist; PA: Physician Assistant; SW: Social Work.

**Table 4:** Quotes that support negative factors.

Discipline	Quote
NU	“Time, focus, logistics.”
NU	“Time is needed to include added curriculum in courses.”
NU	“University departments are not completely integrated.”
NU	“Resistance to change (not personally, but generally) and time constraints for making changes.”
NU	“Difficulty in scheduling issues with various disciplines. Changing the long standing belief that all students need to get through IPE instead of focusing on the purpose of IPE.”
MD	“Lack of cooperation among other professions.”
MD	“Expensive.”
MD	“Lack of school support, particularly financially/administratively and lack of buy-in by some departments.”
MD	“Territory conflicts between different disciplines (e.g., ear, nose, & throat vs oral maxillofacial, orthopedics vs podiatry).”
MD	“Lack of functional care models for truly integrated patient centered care.”
PH	“We are the only health professionals on campus, so it is difficult to engage in IPE.”
PH	“Work load.”
PH	“Currently at non-teaching hospital. Separate nursing, pharmacy, medical schools. No history of IPE. Time commitment to setting up, maintaining, assessing IPE.”
PH	“Time constraints, any bureaucratic issues involved in setting up such a program.”
PH	“Scheduling. Resources.”
PT	“Faculty feel that it will take a lot of work and scheduling is difficult due to different curriculum schedules. To me these are barriers not negative factors.”
PT	“Time. Accessibility.”
PT	“Administrative complexity. Increase work load.”
PT	“Time. Challenging coordinating different programs for availability at same times.”
PT	“Extra time/effort needed to work with faculty from other departments to plan experiences. Difficulty scheduling experiences involving students from more than one program.”
OT	“Scheduling.”
OT	“Politics, competition, steal ideas.”
OT	“Scheduling. People being territorial. More interest on my part than on the part of the other person in another discipline.”
OT	“Lack of time. Difficulty coordinating schedules.”
OT	“Lack of time. Lack of programs to alter schedules to include IPE opportunities. Lack of support by some faculty.”
PA	“Logistics.”
PA	“At times, egos can get in the way of effective teams.”
PA	“Time and effort (both surmountable).”
PA	“Bad attitudes of team members.”
PA	“Difficulty in scheduling multiple disciplines.”
SW	“Silos built into academia.”
SW	“Time. Resource. Coordination.”
SW	“Lack of communication.”
SW	“Different professional values systems could create conflicts.”
SW	“Interdepartmental competition and lack of motivation to collaborate.”

NU: Nursing; MD: Medical Doctor; PH: Pharmacy; PT: Physical Therapist; OT: Occupational Therapist; PA: Physician Assistant; SW: Social Work.

**Table 5:** Positive and negative percentages from each discipline.

Open-Ended Questions	NU (*n* = 191)		MD (*n* = 38)		PH (*n* = 46)		PT (*n* = 50)		OT (*n* = 40)		PA (*n* = 38)		SW (*n* = 36)	
	*n*	%	*n*	%	*N*	%	*n*	%	*n*	%	*n*	%	*n*	%
Positive factors	155	81.2	29	76.3	36	78.3	40	80	36	90	34	89.5	30	83.3
Negative factors	151	79.1	29	76.3	37	80.4	41	82	36	90	34	89.5	31	86.1

NU: Nursing; MD: Medical Doctor; PH: Pharmacy; PT: Physical Therapist; OT: Occupational Therapist; PA: Physician Assistant; SW: Social Work.
